# Objective Structured Clinical Examination to Assess Patient Safety Competencies of Japanese Medical Students: Development and Validation Argument

**DOI:** 10.7759/cureus.73969

**Published:** 2024-11-19

**Authors:** Ikuo Shimizu, Kazumi Tanaka, Jun-Ichirou Mori, Aiga Yamauchi, Sawako Kato, Yuichi Masuda, Yuichi Nakazawa, Hiroyuki Kanno

**Affiliations:** 1 Medical Education, Chiba University Graduate School of Medicine, Chiba, JPN; 2 Center for Medical Education and Clinical Training, Shinshu University School of Medicine, Matsumoto, JPN; 3 Quality and Patient Safety, Chiba University Hospital, Chiba, JPN; 4 Healthcare Quality and Safety, Gunma University Graduate School of Medicine, Maebashi, JPN; 5 Academic Affairs Office, Shinshu University School of Medicine, Matsumoto, JPN

**Keywords:** clinical clerkship, objective structured clinical examination, patient safety based medical education, simulation in medical education, summative assessment, validity argument

## Abstract

Background

The growing emphasis on improving patient safety over the past two decades has received more focus in the undergraduate curricula, and the appropriate assessment of patient safety competencies at graduation is crucial in competency-based medical education. However, there is no valid method for assessing patient safety competencies because current assessment methods in medical education focus less on behavior. The objective structured clinical examination (OSCE) is a method to assess clinical performance and has been implemented by medical schools in Japan for summative assessment at graduation. However, stations with sufficient validity to assess patient safety competencies have not yet been developed. Thus, this study aimed to evaluate, under a contemporary validity framework, an OSCE station for assessing patient safety competencies that students are expected to achieve at graduation from medical schools in Japan.

Methods

A validity argument was conducted using Messick’s validity framework, which includes content, response process, relations to other variables, internal structure, and consequences. First, we applied a modified Delphi study to develop OSCE stations for assessing patient safety competencies based on the national model core curriculum at graduation. The panel survey recruited members who have expertise in clinical education and patient safety. The draft stations simulated various situations associated with patient safety. Final-year medical students then took the OSCE. We analyzed the results of the OSCE, compared the scores with those of the clinical reasoning examination, and evaluated its reliability.

Results

Out of 30 panelists, 22 (73.3%) fully participated in the Delphi rounds. After two Delphi rounds, we established four stations to assess patient safety competencies. They met the content dimension of the validity framework. The OSCE results showed low correlation with clinical reasoning, suggesting that patient competencies cannot be inferred from clinical reasoning. Each station had satisfactory reliability. The entire process minimized possible assessment bias.

Conclusions

The OSCE scenario designed through the modified Delphi study met the five criteria of Messick’s validity framework. The results show that it is a valid strategy for assessing patient safety competencies at graduation.

## Introduction

Despite the widespread attention toward improving patient safety over the past two decades [1], undergraduate medical educators are reluctant to impart knowledge outside clinical disciplines to health profession students, as the World Health Organization (WHO) is concerned [2]. Several educational guides, such as the WHO Patient Safety Curriculum Guide [3] and the Canadian Patient Safety Institute Safety Competency Framework [4], provide suggestions for teaching and learning. The WHO guide includes eleven key learning topics, and the Canadian framework suggests six core competency domains. The WHO also publishes specific patient safety issues in settings where young health professionals are often involved, such as cases of medication errors [5]. As a result, more medical schools have started to implement patient safety curricula [6].

Although assessment is an important part of competency-based education [7], there has been no conclusive opinion about suitable assessment strategies for patient safety competencies [8]. However, according to a systematic review, the assessment of patient safety for medical students focuses less on performance and more on knowledge [9]. Although the WHO promotes the incident reporting system as fundamental to improving patient safety [10], few studies use incident reporting to help acquire competencies and bring about behavioral changes in the undergraduate curriculum [11-13]. From the perspective of assessment theory, examinations that do not directly assess certain skills often result in inadequate measurements [14]. While there are several other methods for self-assessment and workplace-based assessment [15,16], self-assessment can be susceptible to social expectation bias and may cause complications in patient safety [17]. Additionally, when dealing with highly context-dependent events, such as patient safety, there are concerns regarding the consistency of behavioral observations between contexts in the workplace-based assessment [18,19]. As a result, the impacts of patient safety education on attitudinal outcomes have been minimal [9].

To overcome these issues, the objective structured clinical examination (OSCE) can be used because this is an objective and reliable assessment method [20,21]. It has been incorporated into various forms of undergraduate and postgraduate examinations in numerous countries as a clinical competency assessment [21-23]. In Japan, the nationwide OSCE at the end of clinical clerkship (post-CC OSCE) was implemented by the Common Achievement Testing Organization (CATO) [24]. Since students are required to be able to perform these tasks safely upon graduation, university faculty need to assess patient safety competencies relevant to tasks. These competencies are listed in the national model core curriculum (MCC) as “practice medical care with an emphasis on quality and patient safety by giving full consideration to patients’ pain and anxiety and by developing reliable and dependable clinical skills.” The following two sub-items are described in detail: consider and promote patient safety and share information on patient safety [25]. As described by Komasawa et al. [26], the post-CC OSCE in the Japanese undergraduate medical education curriculum is responsible for the summative assessment of competencies, including patient safety.

Currently, the post-CC OSCE assesses only some of the clinical tasks (medical interview, physical examination, oral presentation). For the rest of the competencies, including patient safety, each university is required to assess by means of OSCEs or other assessment methods. Several unassessed competencies must be assessed in the university’s original OSCE or the workplace. However, competencies related to patient safety are more situation-specific in the workplace than those related to clinical procedures. Therefore, the development and validation of OSCE stations that can assess patient safety competencies is a more urgent priority than other clinical procedures.

Several studies have explored assessing patient safety competencies using OSCE [21-23]. However, assessing the development of validated OSCE stations is difficult. First, careful consideration is required to ensure the validity of the OSCE tasks [27]; few patient safety specialists have the ability to construct satisfactory OSCE stations. Second, higher competencies in medical practices and in associated patient safety must be acquired because participation in clinical practice during CC is gradually becoming popular in Japan [28]. However, the existing post-CC OSCE does not cover aspects of patient safety and other summative assessment opportunities. Furthermore, few universities have expert patient safety managers in undergraduate education. For example, only approximately 30% of universities have patient safety managers as curriculum committee members for patient safety education in Japan [29].

The concept of validity has been innovated in recent years. In its conventional concept, subjective aspects were allowed [14]. Face validity, where experts review whether the characteristics of the examination are adequate, is considered the most subjective type of validation. Content validity refers to a more thorough examination of the characteristics and cohesiveness of the assessment, yet it is still subjective because it is based on the narrative opinions of the experts. However, given the nature of patient safety competencies, the subjective and unstructured views of validity are not adequate. Thus, construct validity, the concept regarding how well an assessment reflects what should be assessed must be considered [30].

Under these circumstances, it would be useful for individual universities to create and share validated OSCE stations that meet the required competencies and topics to introduce appropriate patient safety stations in post-CC OSCEs, rather than creating tasks within their universities. If universities start measuring patient safety competencies using validated OSCEs, the patient safety competencies of medical school graduates in Japan can be assessed summatively, thereby improving the safety and quality of healthcare. Considering that OSCE stations have proven to be an effective strategy in assessing the acquisition of practical skills in healthcare, this study’s aim is two-fold: to develop OSCE stations for medical students to assess patient safety competencies, and to identify a valid argument for an OSCE station to assess patient safety competencies. We aim to address the following research questions: What types of stations can we develop to assess patient safety competencies at graduation? To what extent does valid evidence support the use of the patient safety stations as part of the post-CC OSCE?

## Materials and methods

The study was conducted at Shinshu University, Japan, in two phases: development and validation argument. We employed Messick's validity framework [31], which is a current standard for validity arguments in simulation-based assessment [32,33] because in this framework all validity evidence can be included in the construct. The different sources are used to provide a valid argument for the assessment: test content, response process, internal structure, relationships to other variables, and consequences of testing. The “content” aspect of validity examines whether the test content adequately represents the construct it aims to measure [18]. In our context, it involves ensuring that the test covers the required domains of the construct and excludes irrelevant content in patient safety education in the curriculum and competencies. The “response process” investigates the extent to which the test items elicit the intended mental processes [34]. It involves gathering evidence on whether the examination aligns with the construct definition (e.g., blueprint) and the theoretical basis of the test. It also includes the degree of correlation with examinations that measure other competencies. “Internal structure” of validity examines the test’s internal structure. It assesses whether the test items and their relationships align with the theoretical structure of the construct. Cronbach’s alpha is commonly used to show the internal consistency of the structure [35]. The aspect of “relationships to other variables,” or external validity, assesses how well the test results relate to other measures or criteria that are connected to the construct being measured. For example, comparing the cohorts with different clinical experience levels is a valid strategy. The “consequences” aspect of validity addresses the intended and unintended results of test use [36] and examines the impact of test scores, emphasizing the implications of test use and the need for fairness and equity.

Development phase

To ensure the content dimension of Messick’s validity framework and prove that the content of the OSCE aligns adequately with what it is intended to measure, we used a modified Delphi method to develop OSCE stations for assessing patient safety competencies with good content dimensions [37]. A modified Delphi method establishes a consensus on specific issues by collecting opinions from experts in the field interactively [38]. It provides the argument for content validity as it consists of multiple consultation rounds wherein experts indicate their statements, thereby building a consensus. The Delphi study was conducted in accordance with the reporting standards developed in Conducting and REporting of DElphi Studies (CREDES) [39]. The inclusion of different stakeholders in the Delphi method promotes the acceptance of feedback and effective implementation of the stations. Therefore, similar to previous modified Delphi studies on medical education [40,41], we included the stakeholders of undergraduate patient safety education: patient safety managers, educational experts, and clinical educators.

We selected panelists (n = 30) to ensure the representation of the three groups of stakeholders based on their expertise in clinical education and patient safety management: 10 patient safety managers at university hospitals, 10 educational experts, and 10 medical educators who regularly teach medical students at workplaces. We considered recommendations for determining the total number of panelists [42]. The education experts were purposefully selected based on their knowledge of health profession education; those with master’s or higher degrees in health profession education were selected. Patient safety managers at university hospitals regularly take responsibility for patient safety management activities and have more experience in teaching medical students than those in other teaching hospitals. All medical educators were certified and regularly involved in residency training and thus knew the residents’ tasks well. Participation was voluntary; the participants received no rewards, and the data were anonymized.

As a preparation for the Delphi rounds, the first and second authors developed draft OSCE stations based on the blueprint (Table 1). In MCC, it describes patient safety items as “consider and promote patient safety” and “share information on patient safety” [24]. As there are no comparable reports on patient safety OSCE stations, we referred to 15 medical procedures and situations that over half of the medical students might encounter in the workplace (preoperative preparation, writing record documentation, sharing information to health providers, aseptic techniques, surgical procedures, surgical assistance, ultrasound, electrocardiogram, and vital signs check) or simulation training (blood sampling, intravenous line placement, medication process, breast and gynecological examination, tracheal intubation, and basic life support) during their clinical clerkship [43]. From these, we selected situations that fit the categories of “consider and promote patient safety” and “share information on patient safety” in MCC 2016 [24] and had not been sufficiently assessed by the existing OSCEs. As a result, we identified four themes for designing stations: perioperative marking, challenging patient consultation encounters, near-miss during blood sampling, and confirmation of patient information using a double-check procedure.

**Table 1:** Blueprint to assess students' clinical procedures and their selected procedures for station development CAT: common achievement tests; CATO: Common Achievement Testing Organization; CC: clinical clerkship; MCC: model core curriculum; OSCE: objective structured clinical examination

Medical procedures/ situations that students experience during CC		Patient safety in MCC*[24]	Not covered by CAT-OSCEs	Selected procedures for station development
Workplace				
	Preoperative preparation	X	X	X (preoperative marking)
	Writing record documentation	X		
	Sharing information to health providers	X	X	X (actions to a near-miss claim)
	Aseptic technique	X		
	Surgical procedures			
	Surgical assistance		X	
	Ultrasound		X	
	Electrocardiogram			
	Vital signs check			
Simulation				
	Blood sampling	X	X	X (adverse event of venipuncture)
	Intravenous line placement			
	Medication process	X	X	X (double checking for medication)
	Physical exam (breast/ gynecological)	X		
	Tracheal intubation		X	
	Basic life support	X		
We omit the mark in this row if it is recognized as other competencies in MCC.				

We designed the four draft station plans to meet the following four criteria: examinees can complete the task within a test time of seven minutes (consistent with the “short” station defined by CATO); tasks are consistent with the WHO Curriculum Guide and MCC 2016; tasks can be experienced in a typical clinical clerkship program in Japan [24]; and sufficient feasibility is expected (particularly with regards to the availability of equipment in a typical medical school). We used three types of rating scales based on our previous report on OSCE [44]. First, the five steps to be assessed for each item rating scale (IRS) were listed. The items were rated on a six-point scale ranging from one to six in the following stages: preclinical (not allowed to start CC), just starting CC, during CC, completing CC (acceptable to graduate), during residency, and completing residency. Second, the Global Rating Scale (GRS) was rated using a six-point scale ranging from one to six to account for differences between years, with a score of four or higher considered acceptable for the examinees’ academic year.

We sent the online explanation of the draft stations to panelists and asked them to rate each criterion on a five-point Likert scale (1 = unimportant, 2 = of little importance, 3 = neutral, 4 = relevant, and 5 = very relevant) through surveymonkey.com. We then calculated the means and standard deviations of each criterion. We also asked the panelists to change redundant or unnecessary phrases and suggest additional items. Based on the obtained results, the issues were revised and added, and responses were requested again. This process was repeated until all items were aggregated to a mean ≥ 3.5 and a standard deviation ≤ 1.

Validation argument phase

After the “content” dimension of Messick’s validity framework was ensured during the development phase, the remaining validity argument was conducted through the implementation of the OSCE for the final year students at Shinshu University in 2019-2022 [31].

Participants

The patient safety OSCE was administered to sixth-year students who participated in a CC program as part of a six-year undergraduate medical curriculum at Shinshu University. Although testing was mandatory, participation in the study was voluntary, and written informed consent was obtained from those who wished to participate.

Assessment

To minimize possible assessment bias and ensure the “response process” dimension of validity of Messick’s framework, this examination was designed according to the blueprint for assessing clinical years. This examination is based on clinical competencies that students should acquire upon graduating from medical schools in Japan [24]. Of these, medical interviews, physical examinations, clinical reasoning and planning, presentation, writing a patient note, clinical procedures, and discussions with health professionals (i.e., the remaining competencies) were already assessed in the existing post-CC OSCE and regularly assessed in the workplace. Each station took approximately seven minutes to complete. The purpose of the patient safety OSCE was explained to the students in advance, and the results were provided after the examination. To prevent malpractice, students were prohibited from using smartphones or other digital devices. Meeting times and locations for each cohort were separated to prevent contact.

One rater who regularly instructed medical students and residents and was knowledgeable about various levels of performance was present at each station. They had experience with the ratings of previous OSCEs and had received a lecture on each station and the key points of the assessment, as well as a prior review of each station. All performances were video recorded in case of any doubt about the ratings. Assessors who did not have a close relationship with the students were chosen from among faculty members.

All stations were organized by an educational specialist at the Center for Medical Education and Clinical Training who received intensive training in simulation-based education.

According to the practical guidelines of simulation assessment [45], comparing scores to assess different levels of proficiency is a valid strategy to evaluate the “relations to other variables” dimension. We compared the results of the patient safety OSCE station with those of the clinical reasoning assessment as a written test (which answered clinical reasoning questions in the form of a patient note) after obtaining information from medical interviews and physical examinations. The test was chosen because it reflects the clinical knowledge and reasoning skills of the examinees and can assess these competencies objectively and independently of patient safety issues.

Regarding the “internal structure” dimension (the reliability of the scores regarding reproducibility under identical conditions), we assessed internal consistency as the reliability.

Regarding the “consequences of the test” dimension, two types of rating scales were used as written in the development phase. A pass/fail decision was made using the borderline regression method [42]. Each item and a GRS consisting of six levels were scored, and the average score corresponding to three (borderline) of the GRS was considered a passing score. We did not set “immediate fail” errors.

Analysis

“Correlations between measures” were compared using Spearman’s rank correlation coefficient (rs), as the values of these measures were expected to be non-normally distributed based on previous OSCE results (p < 0.05 was considered significant). The internal consistency of each station was assessed using Cronbach’s alpha coefficient, and a coefficient of > 0.7 indicated acceptable reliability [35].

To evaluate the “relationship to other variables,” we compared the IRS scores between the sixth-year (target as summative assessment) and the fifth-year (control) students. T-tests were used to compare the scores across academic years.

Statistical analysis was performed using IBM SPSS Statistics for Windows, Version 27 (Released 2020; IBM Corp., Armonk, New York, United States) and Microsoft Excel 2019 (Microsoft Corporation, Redmond, Washington, United States).

## Results

Development phase

Of the 30 panelists, 22 (73.3%) returned a fully completed questionnaire throughout the process. In the first round of the Delphi method, one station with acceptable ratings was retained. Three of the original four stations revised the task contents or task descriptions based on the panelists’ suggestions.

In the second round, all station plans had standard deviations < 1 (0.30-0.91). As the panelists proposed no additional items and provided no other negative responses, we concluded that no more rounds were necessary and a consensus was reached. Finally, all four stations (Table 2) were completed as they met the criteria.

**Table 2:** Stations developed through the modified Delphi study

No.	Context of the station	Simulated persons
1	Preoperative site marking: Examinee is asked to perform the preoperative marking followed by the confirmation of the surgical site.	Patient, supervising physician
2	Challenging patient encounter: Examinee is asked to manage a complaint about bruising after venipuncture.	Patient
3	Near-miss: Examinee is asked to take a blood sample but the tube is labeled with the name of the wrong patient.	Patient, nurse
4	Double-checking procedure: Examinee is asked to check an intravenous fluid bag before administration by a nurse.	Patient, nurse

The first station comprised the preoperative site marking. The examinee was asked to perform preoperative marking, followed by confirmation of the surgical site. The second station was concerned with challenging patient encounters. The examinee was asked to manage the complaint of bruising after venipuncture. The third station focused on handling a near-miss event. The examinee was asked to collect blood; however, the tube was labeled with the name of the wrong patient. The fourth station performed double-checking procedures. The examinee was asked by a nurse to check the intravenous fluid bag before administration. Since the respective issues were confirmed to be related to the competencies in MCC through the modified Delphi process, the content dimension of Messick’s framework was assured.

Validation argument phase

The four OSCEs were conducted in 2019-2022 for sixth-year students at the end of CC. Students from each year cohort underwent examinations on the same day. There were no missing data for any item in the analysis. Table 3 presents the results of the analysis. The rank correlation with the clinical reasoning task was low, suggesting that patient safety competencies could not be inferred from the written examination of clinical reasoning. The reliability was acceptable for all the tasks. In terms of the “consequences” aspect, two types of rating scales (IRS and GRS) and the decision functioned as a pass/fail result. In accordance with our previous OSCE research [44], the IRS was suitable for assessing students’ abilities, while the GRS was used for summative assessment. The passing rate using the scales was 100%. In terms of the “relationship to other variable” aspect, the IRS scores between the sixth and fifth-year cohorts were compared (Table 4). The IRS scores in each station showed a statistically significant difference. This implies that all stations significantly reflected the differences in experience in clinical settings. Based on these findings, we concluded that the remaining dimensions of Messick’s framework were confirmed.

**Table 3:** Results of objective structured clinical examinations and the dimensions of internal structure and response process Spearman correlation analysis was conducted to evaluate the correlation to clinical reasoning. GRS: global rating scale; IRS: item rating scale; OSCE: objective structures clinical examination

Set	Exam results			Alpha coefficient	Correlations to clinical reasoning [r_s_]
	Scales	Mean	SD		
1 (n = 118)	OSCE (preoperative marking)			0.82	0.11
	IRS	19.31	2.47		
	GRS	4.31	0.71		
	Written clinical reasoning	21.14	2.03		
2 (n = 113)	OSCE (challenging encounter)			0.87	0.13
	IRS	16.35	2.89		
	GRS	3.93	0.49		
	Written clinical reasoning	20.94	2.37		
3 (n = 135)	OSCE (near-miss)				0.20*
	IRS	20.0	4.81	0.90	
	GRS	4.18	1.09		
	Written clinical reasoning	18.54	2.59		
4 (n = 130)	OSCE (double-checking)			0.98	0.11
	IRS	19.8	8.62		
	GRS	4.35	0.77		
	Written clinical reasoning	18.73	1.52		
* p < .05					

**Table 4:** Comparison of IRS scores between the final-year (target) and the fifth-year (control) cohorts CI: confidence interval; IRS: item rating scale

	6th-year (target)		5th-year (control)		p
	Mean	SD	Mean	SD	[95% CI]
Preoperative marking	19.31	2.47	16.72	2.75	<0.01*
(6th) n = 118, (5th) n = 114					[1.91, 3.27]
Challenging encounter	16.39	2.81	15.00	3.85	0.04*
(6th) n = 99, (5th) n = 15					[0.03, 3.17]
Near-miss	20.00	4.81	17.19	5.63	<0.01*
(6th) n = 135, (5th) n = 131					[2.29, 4.51]
Double-checking	19.8	8.62	12.27	2.13	<0.01*
(6th) n = 130. (5th) n = 114					[2.61, 3.51]
*p < .05					

## Discussion

The acquisition of appropriate patient safety competencies is an important outcome of general clinical training and is directly related to improving the quality of care. However, validated methods for assessing patient safety competencies are not yet being constructed so far [46]. Assessments are often conducted as written or oral examinations, with little or no practical assessment. Workplace-based assessments are slowly becoming widespread [47]; however, these assessments are often descriptive and require numerous assessment opportunities [48]. Self-assessment skills are accurate only in some situations, as Eva and Regehr [49] argue, and should be used along with valid external assessments, such as OSCE, for assessing medical competencies. Given the growing interest in teaching patient safety in undergraduate medical education curricula, this study validated the OSCE to assess patient safety competencies by developing OSCE stations with satisfactory content evidence and analyzing the other dimensions based on Messick’s validity framework. There have been few validation studies that use the standard Messick’s validity framework in simulation assessment, which relies on surface validity and other factors [46,48]. As is the case in many countries, including Japan, some competencies and tasks required at graduation have not been properly assessed. The students are assessed through a written or oral exam with little or no practical skills assessment. Residents are sometimes assessed using real patient cases. However, these assessment opportunities are often descriptive and non-standardized. Simulation tools are considered better for assessing practical skills than written examinations [46]; OSCE scenarios assess practical skills in a standardized and reproducible manner and provide comparable results. Although the OSCE has already been implemented in some clinical areas (e.g., anesthesiology and family practice), using Messick’s validity framework, the results show that implementing the validated OSCE stations for patient safety education is reasonable. In terms of “internal structure,” all stations demonstrated high reliability, which is an advantage of the OSCE over workplace-based assessments [50]. One study in postgraduate clinical training found that more than 10 assessors per physician were required to achieve a reliable assessment in many workplace-based assessment tools, significantly reducing the feasibility of using them for high-stakes assessments [15]. Another study found that a combination of multiple assessment methods is necessary to maintain accuracy [51]. Although OSCE is a resource-intensive assessment method, feasibility and reliability are also traded for workplace-based assessment. In terms of “content,” all stations created in this study were considered appropriate because they are associated with the tasks and competencies expected at graduation (Table 2) and improved during the clinical clerkship (Table 4). Furthermore, through the formulation of stations, we were able to embody clinical settings fraught with the types of patient safety risks that medical students often face in CC. These environments included OSCE tasks and situations wherein the supervising physician collaborated with the students to maintain patient safety. They can be recognized by the learners as OSCE simulated "shows-how” and as “does”; that is, situations that should be learned through actual actions [25]. An explicit discussion on patient safety in the context of these situations will provide a valuable and enriching opportunity [52]. Without such opportunities, structural gaps in the curriculum may continue to limit new graduates’ abilities to improve care and prevent errors [53]. Therefore, learning opportunities should not be hidden, even with an OSCE as a summative assessment; they should be proactive and provide learning opportunities for this type of scenario.

The OSCE results correlated with levels of expertise, meeting the “relation to other variables.” This result can be explained as measuring a general aspect of competence that improves through the clinical clerkship since the IRS is a measure of proficiency in each task. Among all OSCE stations, the challenging encounter task had a small, albeit significant, point difference between the fifth and sixth-year cohorts. This may be because of the limited opportunity for students to experience dealing with difficult encounters.

The “response process” section of the framework was also evaluated and formulated. Other clinical reasoning competencies correlated slightly with safety station performances. This suggests that the OSCE stations developed in this study can measure different competencies from clinical reasoning. Patient safety OSCEs cannot be replaced by clinical reasoning tests, and vice versa. However, a significant difference in the correlation coefficient was observed in Station 3 (response to a near-miss), although it was small. This indicates that this station might include a reasoning process that lists several possibilities related to the situation. If the examinees came up with the possibility that the examination settings were incorrect, they might have had to distinguish that the error in the scenario was a simulation and a setup error in such a station. Careful arrangement of the equipment and announcements will be required to assess patient safety competencies of reasoning.

Compared with the workplace-based assessment, the OSCE is easier to standardize; training the assessors is easy as well [54]. In our OSCE stations, we applied the combination of GRS and IRS based on our previous study [44]. The use of OSCEs scored on a GRS was consistent with the following two important trends: increased emphasis on professional competencies (including patient safety) and the perceived value of learning skills in an integrated manner [55]. We employed a borderline group method using multiple measures, considering that the response process could be well-maintained because it allowed for a clear summative decision. Raters with sufficient experience were engaged in the assessment after training. However, a convergent assessment by multiple raters will decrease feasibility [56]. Our results indicate that OSCE stations designed to assess patient safety competencies required by Japanese medical students at graduation can achieve station scores with sufficient validity.

Notably, patient safety competencies are common to physicians and many other health professions, such as nurses; these may be transferable as an assessment of their competencies. This could also be of interest to learning programs that seek greater opportunities for interprofessional education. Simultaneously, providing feedback and debriefing opportunities on OSCEs can maximize opportunities for interprofessional interactions. Reports on physicians and nurses taking the same OSCE to assess limited patient safety competencies have existed in the past [57], and it is expected that a similar study on broader patient safety competencies will be conducted in the future based on this study.

Limitations

First, we note that in this study, information on internal structure is limited. We could not report the inter-station or inter-rater reliability. However, many high-stakes OSCEs are administered by a single rater, and structured rating scales can reduce reliability decay [18]. Additionally, although we only assessed raters belonging to the same context, information about the internal structure was enriched by a diverse set of raters. Second, the themes of each of the stations we constructed were different, and a summative assessment combined with the workplace-based assessment and this OSCE would provide more useful information than assessing only with the OSCE [27]. The theoretical combination of multiple assessment methods, such as programmatic assessment [56], will reinforce our findings on how to combine patient safety OSCE and workplace-based assessment. Third, information regarding learners’ response processes is scant. For example, learning from summative assessments has not been explored. Although validated in this study, summative assessments, if authentic, have a learning effect. The learning of students from this test should be explored in the future. Fourth, this study was conducted in a single country. As mentioned in the introduction, patient safety is a context-dependent issue. In addition, we used data from medical students in Japan to develop the draft stations in the Delphi process. Therefore, while we can argue that patient safety competencies are assessed by the OSCE, whether the stations we used would be useful in other countries requires further validation.

## Conclusions

Patient safety competencies at graduation can be assessed using the validated OSCE stations. Consensus-building methods allow for the creation of scenarios that ensure authenticity and feasibility. The value of this study lies in the establishment of simulation-based assessment for medical students’ patient safety competencies. In the future, these could be used to assess the competencies of other health professions.
